# The Effects of Cutaneous Fatty Acids on the Growth of *Pseudogymnoascus destructans*, the Etiological Agent of White-Nose Syndrome (WNS)

**DOI:** 10.1371/journal.pone.0153535

**Published:** 2016-04-12

**Authors:** Craig L. Frank, Melissa R. Ingala, Rebecca E. Ravenelle, Kelsey Dougherty-Howard, Samuel O. Wicks, Carl Herzog, Robert J. Rudd

**Affiliations:** 1 Department of Biological Sciences, Fordham University, Louis Calder Center, P.O. Box 887, Armonk, NY, 10504, United States of America; 2 Environmental Science Program, Fordham University, LH 400, Bronx, NY, 10458, United States of America; 3 New York State Department of Environmental Conservation, 625 Broadway, Albany, NY, 12233, United States of America; 4 New York State Department of Health, Wadsworth Center, Albany, NY, 12201, United States of America; CSIRO, AUSTRALIA

## Abstract

White Nose Syndrome (WNS) greatly increases the over-winter mortality of little brown (*Myotis lucifugus*), Indiana (*Myotis sodalis*), northern (*Myotis septentrionalis*), and tricolored (*Perimyotis subflavus*) bats. It is caused by a cutaneous infection with the fungus *Pseudogymnoascus destructans* (*Pd*). Big brown bats (*Eptesicus fuscus*) are much more resistant to cutaneous infection with *Pd*, however. We thus conducted analyses of wing epidermis from hibernating *E*. *fuscus* and *M*. *lucifugus* to determine their fatty acid compositions, and laboratory *Pd* culture experiments at 4.0–13.4°C to determine the effects of these fatty acids on *Pd* growth. Our analyses revealed that the epidermis of both bat species contain the same 7 fatty acid types (14:0, 15:0, 16:0. 16:1, 18:0, 18:1, & 18:2), but the epidermis of *M*. *lucifugus* contains: a) more stearic (18:0) acid, b) less palmitoleic (16:1) acid, c) less myristic (14:0) acid, and, d) less oleic (18:1) acid than that of *E*. *fuscus*. The growth of *Pd* was inhibited by: a) myristic and stearic acids at 10.5–13.4°C, but not at 4.0–5.0°C, b) oleic acid at 5.0–10.6°C, c) palmitoleic acid, and, d) linoleic (18:2) acid at 5.0–10.6°C. One set of factors that enables *E*. *fuscus* to better resist cutaneous *P*. *destructans* infections (and thus WNS) therefore appears to be the relatively higher myristic, palmitoleic, and oleic acid contents of the epidermis.

## Introduction

About 47 bat species occur in North America, and most hibernate during winter [1]. White-nose Syndrome (WNS) is an emergent disease that has killed over 6,000,000 bats in the eastern USA and Canada. Mass mortality with WNS was first found at 6 caves in central NY State during the winter of 2006–07. WNS has since spread to > 190 bat hibernation sites located in 27 U.S. states and 5 Canadian provinces. WNS leads to over-winter mortality rates as high as 98% for 4 of the 6 species that over-winter in the northeast: little brown (*Myotis lucifugus*), Indiana (*Myotis sodalis*) northern (*Myotis septentrionalis*) and tricolored (*Perimyotis subflavus*) bats [2]. A white fungus associated with WNS has been identified as *Pseudogymnoascus destructans* (*Pd*), and it grows on the muzzle, wings, and ears of affected bats during hibernation [3, 4]. Histological analyses of affected *M*. *lucifugus*, *M*. *septentrionalis*, and *P*. *subflavus* revealed that *Pd* hyphae penetrate both the epidermis and dermis, replacing hair follicles, sebaceous and sweat glands [3, 5] during hibernation. The optimal temperature for the growth of *Pd* is 12.5–15.8°C [6]. Laboratory experiments reveal that cutaneous infection with *Pd* is the cause of death in WNS-affected bats [7, 8].

The hibernating bat species found in North America feed almost entirely on insects when active [9–11]. During late summer/early fall, the body fat content of little brown bats (*M*. *lucifugus*) increases from 7 to 27% body mass [12, 13]. Fat is the primary energy source utilized during mammalian hibernation [14, 15]. Mammalian torpor involves the regulation of body temperature (T_b_) at a new and substantially lower level, with a new critical minimum T_b_ maintained [16]. Hibernators do not remain torpid throughout the hibernation season; instead bouts of torpor last for days, interrupted by brief (< 3 h for bats) periods of high metabolic rate and T_b_, known as arousal episodes that account for 80–90% of all energy (fat) utilized during hibernation [16, 17].

Field studies indicate that cutaneous infection with *P*. *destructans* causes mortality through the disruption of torpor patterns during hibernation. Infected bats arouse more frequently from torpor during hibernation, which leads to a premature depletion of body fat reserves prior to the availability of food, and subsequent death [18, 19]. Infection with *P*. *destructans* does not produce mortality during hibernation in all bat species. Examination of hibernation sites in Europe revealed *P*. *destructans* growing on the muzzles of 5 different European bats (*Myotis dasycneme*, *M*. *myotis*, *M*. *duabentonii*, *M*. *brandtii*, and *M*. *oxygnathus*) during torpor. Mass deaths were not observed at these sites, however, [20, 21]. Histological analyses of infected *M*. *myotis* revealed that the hyphae of *P*. *destructans* do not extend beyond the epidermis of this bat species, even after several months of hibernation [22]. A field study conducted by our laboratory demonstrated that big brown bats (*Eptesicus fuscus*) hibernating in the same New York mines where *M*. *lucifugus* develop severe *Pd* infections (and WNS): a) have torpor bouts of normal duration, b) do not develop cutaneous *Pd* infections, and, c) usually survive the hibernation period with some body fat reserves remaining [23].

These observations lead to the question: *What are the epidermal properties that enable some bat species to better resist cutaneous infection by P*. *destructans*? The epidermis is composed chiefly of specialized squamous epithelial cells named keratinocytes that occur in 4 distinct strata; they are produced in the deepest stratum (the stratum basale), and migrate to the top stratum (the stratum corneum) as they mature [24, 25]. The lipids of the stratum corneum are a mixture of compounds from both the extracellular matrix secreted by the keratinocytes, and sebum produced by the sebaceous glands. The extracellular matrix contains free sphingosine bases, ceramides, cholesterol, and free fatty acids (FFAs), whereas the sebum is composed of triacylglycerols, diacylglycerols, FFAs, wax esters, squalene, and cholesterol [26–28]. Free fatty acids account for almost half of all lipids found in the mammalian epidermis [29], and consist of both saturated and unsaturated fatty acids ranging from 12 to 20 carbon atoms in length [24, 25]. Some of these saturated and unsaturated FFAs have potent antimicrobial properties in laboratory experiments [29]. We thus predicted that: a) some of the fatty acids found in the wing epidermis of bats inhibit the growth of *Pd*, and, b) the wing epidermis of bat species susceptible to cutaneous infection with *Pd* have relatively lower contents of the fatty acid types that inhibit the growth of *Pd* than those of bat species that are resistant to *Pd*. These predictions were tested by first analyzing the fatty acid profiles of the wing epidermis from free-ranging little brown (*M*. *lucifugus*), and big brown (*E*. *fuscus*) bats. We then conducted laboratory growth experiments with colonies of *P*. *destructans* cultured on media varying in the contents of the same FFA types found in epidermis of both bat species.

## Materials and Methods

### Wing Skin and Epidermis Analyses

This portion of the present study involved wing skin samples collected from the carcasses of *M*. *lucifugus* and *E*. *fuscus* that were captured and sacrificed using an Isoflurane overdose for 2 previous studies [19, 23], thus no additional bats were used for the present study. The previous studies on *M*. *lucifugus* and *E*. *fuscus* [19, 23] were conducted in strict accordance with recommendations listed in the Guide for the Care and Use of Laboratory Animals of the National (US) Institutes of Health. The protocols were approved by the Fordham University Institutional Animal Care and Use Committee (protocol numbers CF11-03, 12–01, and 12–02). Protocols were also approved by the New York State Department of Health Institutional Animal Care and Use Committee. The capture of live bats in NY was also conducted under a Scientific License to Collect (#1373) issued by the New York State Department of Environmental Conservation.

We analyzed the wing epidermis from 6 free-ranging *E*. *fuscus* and 5 *M*. *lucifugus* previously collected during hibernation for total (free and ester-bound) fatty acid composition. Individuals of both species were collected from the same 2 adjacent abandoned mines located in Ulster County, New York (N41°50.64’, W74°04.92’). All bats were collected on the same day while torpid during the middle of the hibernation period, immediately sacrificed, and stored at -20°C [19, 23]. Small (2–4 cm) samples of the skin were collected from both wings of each bat carcass prior to their use in the previous studies. The epidermis was isolated from these samples using the techniques of Law *et al*. [30]. All lipids were then extracted from the epidermis using a chloroform/methanol procedure [31]. They were trans-esterified using 1.0 methanolic HCl, producing fatty acid methyl esters [32]. Fatty acid methyl esters (FAMEs) were then identified and quantified using a Model 5890 gas-liquid chromatograph (Hewlett Packard, Palo Alto, CA, USA) fitted with a Model DB-23 capillary column (J&W Scientific Inc., Folsom, CA, USA) that is 30 m long. The column was initially held at 110°C for 3 min., then raised to 160°C at 20°C/min., and finally brought to 210°C at a rate of 4°C/min. The carrier gas was helium flowing at a rate of 30 cm^3^/min. Fatty acid methyl esters were detected using a Flame Ionization Detector (FID). Mixtures of known FAME standards (NHI-C, GLC -10, GLC, 40, GLC-50, GLC-70, and GLC-80) obtained from Supelco, In., (Bellefonte, PA, USA) were also analyzed using this apparatus and protocol in order to accurately obtain the retention time of each FAME type. This system thus permitted the identification and quantification of all fatty acids types that were 12 to 22 carbon atoms in length [33].

The wing epidermis of 2 additional *M*. *lucifugus* groups previously collected [19, 23] just prior to the hibernation period (October), and during late hibernation (March), was also analyzed for fatty acid composition in order to determine the effects of hibernation on wing lipid composition. Twelve adult *M*. *lucifugus* were collected from a WNS-affected mine in Albany County, New York (N42°38.80’, W73°44.02’), while euthermic. Eight more adult *M*. *lucifugus* were collected from a cave in Carter County, Kentucky (N38°18.01’, W83°10.44’) during March, where WNS did not occur during that hibernation season (2007–08), while they were torpid. Bats were collected from this site in order to obtain wing skin free of *P*. *destructans* mycelia. All bats were sacrificed immediately upon capture, and stored at -20°C for later analyses. The isolated epidermis from one wing of each bat carcass was then analyzed for fatty acid composition using the techniques described previously. Samples of the skin (epidermis & dermis) from the other wing from each bat carcass was analyzed for total crude lipid content (% dry mass) by extraction with petroleum ether using a Soxhlet apparatus [34]. Calculations using the body compositions previously reported for these same bats [19] and the wing lipid fatty acid contents published for bats [35] reveals that the total fatty acid content of the *M*. *lucifugus* wing epidermis averaged 78.9 mg/g dry matter, whereas that for the *E*. *fuscus* epidermis was 99.7 mg/g dry matter. These values were used to determine the concentration of each fatty acid type for each epidermal sample in mg fatty/g dry matter.

### Laboratory Growth Experiments with *P*. *destructans*

Isolates of *P*. *destructans* used in this study were previously cultured from affected bats in NY during February 2008 (American Type Culture Collection, ATCC MYA-4855). Starter cultures for the growth experiments were initiated from frozen glycerol culture stocks by transfer to Sabouraud dextrose agar (SDA) plates and incubation at 12°C for 5 weeks. For each experimental media/temperature treatment examined, starter culture material was transferred using a sterile disposable inoculating needle to 3 evenly spaced locations on the surface of each of 5–12 experimental SDA plates, yielding 15–36 replicates for each experimental media/temperature combination. The skin temperature (T_skin_) of torpid *M*. *lucifugus* is normally 5–7°C during hibernation [18, 36], whereas the T_skin_ of torpid *E*. *fuscus* in a similar area was 7.5–13.3°C [23]. Experiments were thus conducted at low (4.0–5.1°C) and high (10.5–13.4°C) ambient temperatures to simulate conditions on the skin of torpid *M*. *lucifugus* and *E*. *fuscus*. Each group of plates was incubated for 40–50 d, to the point at which adjacent colonies began to overlap. Growth was quantified by measuring the total surface area visible for each colony. The surface area of each colony (mycelium) was measured at 7 d intervals by capturing digital images of each culture plate with an UVP Chromato-Vue (Upland, CA, USA) model C-75 viewing cabinet. We then calculated the surface area of each photographed colony using ImageJ Version 1.34S software (NIH, Bethesda, MD, USA). We began measurements of colony areas once they became visible to the unaided eye, which was after 12–14 d of incubation at 10.5–13.4°C, and after 20–21 d of incubation at 4–5.0°C. Inoculated plates were sealed inside plastic containers with sterile paper towels moistened with sterile water at the bottom in order to maintain a relative humidity of ~100% during incubation. A single iButton model DS1922L logger (Maxim Semiconductor, Dallas, TX, USA) was placed inside each container to measure ambient temperature (T_a_) at 1 h intervals throughout incubation.

Five different experiments were performed, each of which involved 3 different types of modified SDA media. The compositions of the media types used in each experiment are listed in Table 1. The control treatments used in Experiments 1, 2, and 3 consisted of a group of plates that contained SDA media only. All other treatments in experiments 1–4 were SDA media with enough of either myristic (14:0), palmitic (16:0), palmitoleic (16:1), stearic (18:0), oleic (18:1), or linoleic (18:2) acid added to bring the total FFA concentration to 0.5–1.0% wet mass. This is within the range of concentrations of individual fatty acid types found in the wing epidermis of *M*. *lucifugus* when the fatty acid contents listed are converted to % wet (live) mass using the skin crude lipid contents obtained (see Results). FFAs account for 40% of all lipids in the wing skin of bats [36], and mammalian skin has a water content of 65% wet mass [37]. Consequently, the least abundant fatty acid (18:0) is 0.1% wet mass, whereas the most abundant fatty acid (16:0) accounts for 2.3% wet mass, of the wing epidermis from *M*. *lucifugus*.

**Table 1:** SDA media and ambient temperature (T_a_) in the *P*. *destructans* growth experiments.

Experiment	Media Treatments	Low T_a_ (°C) N		High T_a_ (°C) N	
1	Control	4.0	21	13.4	21
	0.5% Myristic Acid (14:0*)	4.0	18	13.4	18
	0.5% Palmitic Acid (16:0)	4.0	18	13.4	18
2	Control	5.0	36	10.5	30
	0.5% Stearic Acid (18:0)	5.0	24	10.5	30
	0.5% Oleic Acid (18:1)	5.0	27	10.5	34
3	Control	NONE		10.5	15
	1% Palmitoleic Acid (16:1)	NONE		10.5	21
	1% Oleic Acid (18:1)	NONE		10.5	24
4	1% Stearic Acid (18:0)	5.0	27	10.6	34
	1% Oleic Acid (18:1)	5.0	24	10.6	24
	1% Linoleic Acid (18:2)	5.0	24	10.6	24
5	0.25% Myristic Acid (14:0)	5.0	36	10.5	21
	1.0% Myristic Acid (14:0)	5.0	33	10.5	30
	2.0% Myristic Acid (14:0)	5.0	21	10.5	36

* The number left of the colon indicates the number of carbon (C) atoms, the number to the right denotes the number of C-C double bonds [38].

Experiments 1, 2, 3 and 4 were conducted to determine the relative effects of the most common fatty acids found in the wing skin on the growth of *Pd*, whereas Experiments 5 was conducted to determine the relative effects of free fatty acid concentration per se on *Pd* growth. Myristic acid was selected for use in Experiment 5 since the previous experiments revealed that the effects of this FFA on *Pd* growth were moderate. All fatty acids added to SDA media were in the free (unbound) form, > 99% pure, and obtained from the Sigma-Aldrich Chemical Co. (St. Louis, MO, USA). The saturated fatty acids were added after the media was brought to a boil during preparation, just prior to being autoclaved. The unsaturated fatty acids were added to the media after it was autoclaved and had cooled to 60–70°C, just prior to the pouring of plates.

Mean colony areas at the end of each *Pd* growth experiment were compared between media treatments within the same T_a_ group using a one-way ANOVA (General Linear Models) procedure in conjunction with Tukey’s Highly Significant Difference (HSD) Test. Mean fatty acid compositions (both % of all fatty acids and mg/tissue) were compared between the 2 bat species/groups using the Student’s *t*-test. All statistical methods were performed using SYSTAT version 12.0 software. Significance level was set at P < 0.05 for all statistical tests.

## Results

### Wing Skin and Epidermis Analyses

Seven different fatty acid types were found in the lipids of the wing epidermis from both bat species (Table 2), 4 of which were saturated. The wing epidermal lipids of *E*. *fuscus* had more than twice the mean myristic acid (14:0) level of that from *M*. *lucifugus*, when calculated on a % of all fatty acids basis (t = 2.04, df = 9.0, p = 0.038), and a greater mean oleic (18:1) acid level (Table 2) as well (t = 3.722, df = 9, p = 0.005). In contrast, the wing epidermal lipids of *M*. *lucifugus* had a mean pentadecanoic acid (15:0) level that was 7 times greater (t = -2.041, df = 9, p = 0.036) and mean stearic acid (18:0) level that was over 3-fold greater (Table 2) than those from *E*. *fuscus* (t = - 7.139, df = 9, p < 0.001). The wing epidermal lipids of these species did not significantly differ in either mean palmitic (16:0), palmitoleic (16:1), or linoleic (18:2) acid levels (Table 2), however, (t = -1.614, p = 0.14, t = 1.581, p = 0.15, and, t = -0.922, p = 0.38, respectively, df = 9 at each level).

**Table 2:** Mean (± SE) fatty acid compositions of wing epidermal lipids and tissues.

Fatty Acid Type	Symbol*	(% of all fatty acids in lipids)		(mg/g dry epidermis)	
		*M*. *lucifugus*	*E*. *fuscus*	*M*. *lucifugus*	*E*. *fuscus*
Myristic Acid	14:0	5.8 ± 1.5	11.7 ± 2.5**	4.5 ± 1.2	11.7 ± 2.5**
Pentadecanoic Acid	15:0	1.4 ± 0.6	0.2 ± 0.2**	1.2 ± 0.5	0.2 ± 0.2
Palmitic Acid	16:0	34.6 ± 3.2	27.1 ± 3.3	27.3 ± 2.5	27.0 ± 3.3
Palmitoleic Acid	16:1	12.4 ± 1.0	17.8 ± 3.0	9.8 ± 0.8	17.7 ± 3.0**
Stearic Acid	18:0	10.5 ± 0.4	3.0 ± 0.9**	8.3 ± 3.5	3.0 ± 0.9**
Oleic Acid	18:1	14.1 ± 0.8	27.6 ± 3.2**	11.1 ± 6.4	27.5 ± 3.2**
Linoleic Acid	18:2	9.6 ± 0.6	7.2 ± 2.3	7.5 ± 5.1	7.2 ± 2.3

*The digit left of the colon is the number of C atoms; right is the number of C-C double bonds [38].

**Significantly different from the corresponding *M*. *lucifugus* mean at p < 0.05. N = 5 at each level for *M*. *lucifugus* means, N = 6 for *E*. *fuscus* means.

Calculating wing fatty acid contents on a mg/g dry epidermis revealed a similar trend. The wing epidermis of *E*. *fuscus* had over twice the mean myristic acid content (Table 2) of that from *M*. *lucifugus* (t = 2.40, df = 9, p = 0.04), as well as greater mean palmitoleic (t = 2.37, df = 9, p = 0.04) and oleic (t = 5.011, df = 9, p = 0.003) acid contents. The epidermis of these two bat species did not significantly differ (Table 2) in mean pentadecanoic (t = -1.858, df = 9, p = 0.096), palmitic (t = -0.073, df = 9, p = 0.943), or linoleic (t = -0.151, df = 9, p = 0.88) acid contents, however.

The same 7 fatty acid types were also found in the wing epidermal lipids of the 2 additional groups of *M*. *lucifugus* collected both just prior to hibernation, and during late hibernation (Table 3). The wing epidermal lipids of *M*. *lucifugus* collected just prior to hibernation had significantly greater mean myristic (t = 1.852, df = 18, p = 0.04) stearic (t = 2.387, df = 18, p = 0.028), and linoleic (t = 1.865, df = 18, p = 0.039) acid levels than those of *M*. *lucifugus* collected during late hibernation, when calculated on a % of all fatty acids basis (Table 3). The wing epidermal lipids from *M*. *lucifugus* collected just prior to hibernation had lower mean pentadecanoic (t = - 2.624, df = 18, p = 0.017), palmitoleic (t = -2.750, df = 18 p = 0.013) and oleic (t = - 2.332, df = 18, p = 0.032) acid levels (Table 3) than those collected during late hibernation, in contrast. These two lipid groups did not significantly differ in mean palmitic (t = -1.277, df = 18, p = 0.218) acid levels (Table 3), however. The mean (± SE) crude lipid content of the entire wing skin from the pre-hibernation *M*. *lucifugus* was 46.9 ± 5.1% of dry mass, and was greater (t = 2.688, df = 14, p = 0.018) than the mean crude lipid content of 31.9 ± 2.1% observed for the wing skin collected during late hibernation.

**Table 3:** Mean (± SE) fatty acid compositions of *M*. *lucifugus* epidermal lipids and tissues.

Fatty Acid Type	Symbol*	(% of all fatty acids in lipids)		(mg/g dry epidermis)	
		Pre-Hib.	Late Hib.	Pre-Hib.	Late Hib.
Myristic Acid	14:0	8.0 ± 0.9	4.8 ± 1.6**	14.9 ± 1.7	6.1 ± 2.0**
Pentadecanoic Acid	15:0	2.9 ± 0.9	6.7 ± 0.2**	5.4 ± 1.7	8.5 ± 1.5
Palmitic Acid	16:0	30.0 ± 2.7	35.0 ± 3.0	55.7 ± 5.2	44.7 ± 3.9
Palmitoleic Acid	16:1	19.2 ± 2.4	27.7 ± 1.2**	36.0 ± 4.4	35.3 ± 1.5
Stearic Acid	18:0	5.3 ± 1.1	1.9 ± 0.8**	9.9 ± 2.1	2.4 ± 1.1**
Oleic Acid	18:1	14.9 ± 1.3	19.0 ± 1.1**	28.0 ± 2.5	24.2 ± 1.4
Linoleic Acid	18:2	6.6 ± 1.0	3.5 ± 1.4**	12.4 ± 1.9	4.5 ± 1.7**

*The digit left of the colon is the number of C atoms; right is the number of C-C double bonds [38].

**Significantly different from the corresponding pre-hibernation mean at p < 0.05. N = 12 at each level for pre-hibernation means, N = 8 for late hibernation means.

We calculated the total fatty acid content of the wing epidermis from *M*. *lucifugus* collected just prior to hibernation to be 188 mg/g dry matter, whereas that for epidermis from *M*. *lucifugus* collected during late hibernation was estimated to be 128 mg/g dry matter, assuming that 40% of all epidermal lipids are fatty acids. Monoacylglycerols account for 2–4% of all lipids found in the wing epidermis of bats, whereas FFAs account for 17–40%. These tissues contain only trace amounts triacylglycerols and phospholipids, however [36]. Fatty acids account for about 80% of the mass of monoacylglycerols [38]. Fatty acids in both the free and monoacylglycerol (bound) form thus account for about 40% of this lipids found in the wing epidermis of bats.

The mean mystic acid content of the epidermis collected prior to hibernation is more than twice that (Table 3) of the epidermis from bats collected during late hibernation (t = 3.276, df = 18, p = 0.004) on a mg/g dry epidermis basis, and the epidermis of the pre-hibernation group had greater mean stearic (t = 2.710, df = 18, p = 0.014) and linoleic (t = 2.890, df = 18, p = 0.01) contents as well (Table 3). These epidermal groups did not significantly differ in either mean pentadecanoic (t = -1.299, df = 18, p = 0.21), palmitic (t = 1.546, df = 18, p = 0.14), palmitoleic (t = 0.122, df = 18, p = 0.90), or oleic (t = 1.143, df = 18, p = 0.268) acid contents (Table 3), however.

### Laboratory Growth Experiments with *P*. *destructans*

**Fig 1:**
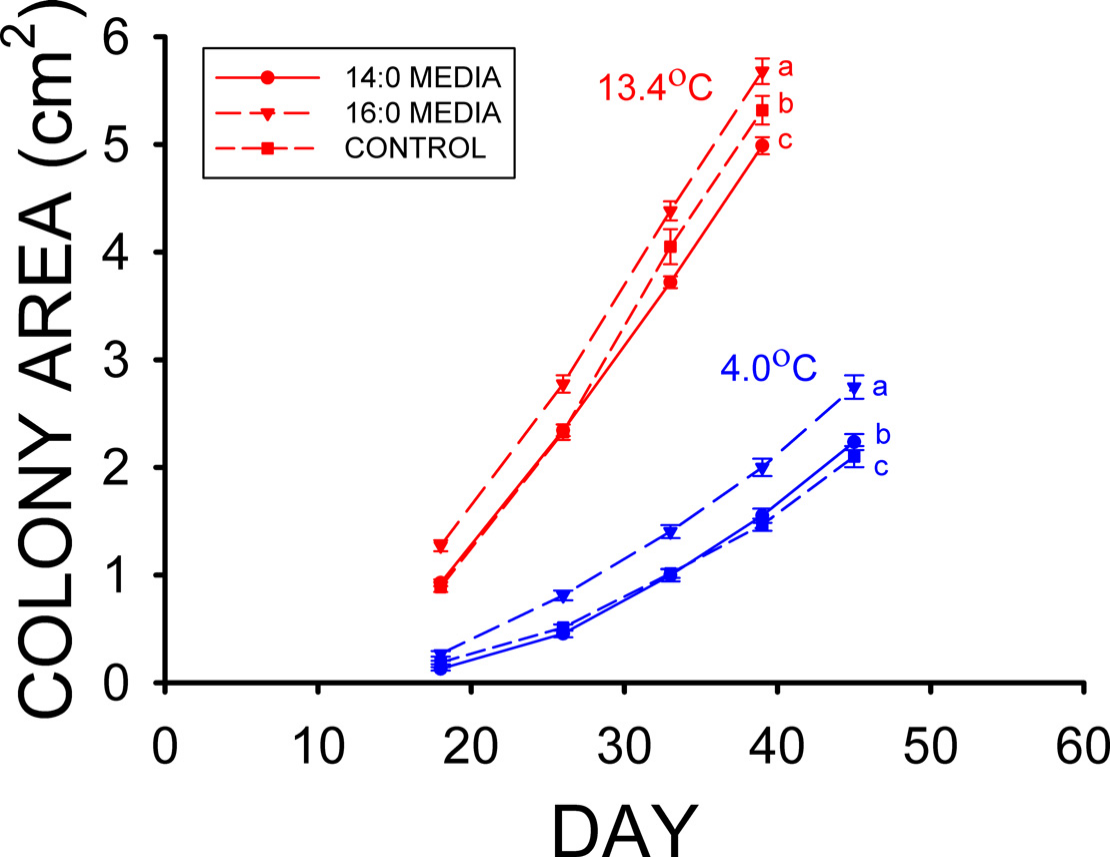
Mean (± SE) surface areas of *Pseudogymnoascus destructans* colonies at various stages of growth on control (squares), 0.5% myristic (14:0) acid (circles), and 0.5% palmitic (16:0) acid (triangles) SDA media at T_a_ = 4 (blue symbols) and 13.4°C (red symbols). Means within the same T_a_ treatment sharing a common lower-case letter are not significantly different at the p < 0.05 level.

The mean colony (mycelium) area of the 0.5% myristic (14:0) acid treatment was less (Fig 1) than those of the control and 0.5% palmitic (16:0) acid treatment (F_2,60_ = 9.412, p < 0.001) in the high T_a_ group of the first experiment. The control plates had a smaller mean colony area than the palmitic acid treatment at the high T_a_ by the end of the first experiment as well (Fig 1). Growth at the low T_a_ for 45 d resulted in a greater mean colony area for the 0.5% palmitic acid media (Fig 1) treatment (F_2,57_ = 25.143, p < 0.001), and the mean colony area for myristic acid treatment was also greater than that of the control.

**Fig 2:**
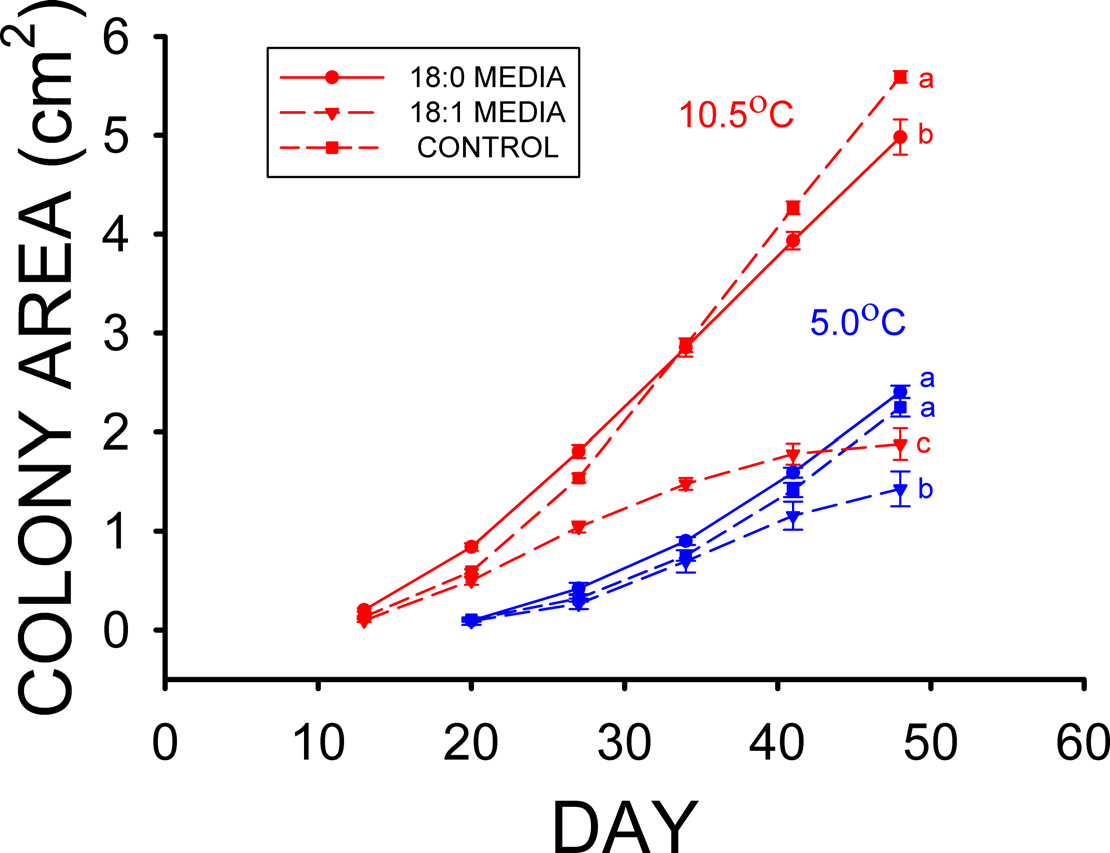
Mean (± SE) surface areas of *Pseudogymnoascus destructans* colonies at various stages of growth on 0.5% stearic (18:0) acid (circles), 0.5% oleic (18:1) acid (triangles), and control (squares) SDA media at T_a_ = 5.0 (blue symbols) and 10.5°C (red symbols). Means within the same T_a_ treatment sharing a common lower-case letter are not significantly different at the p < 0.05 level.

**Fig 3:**
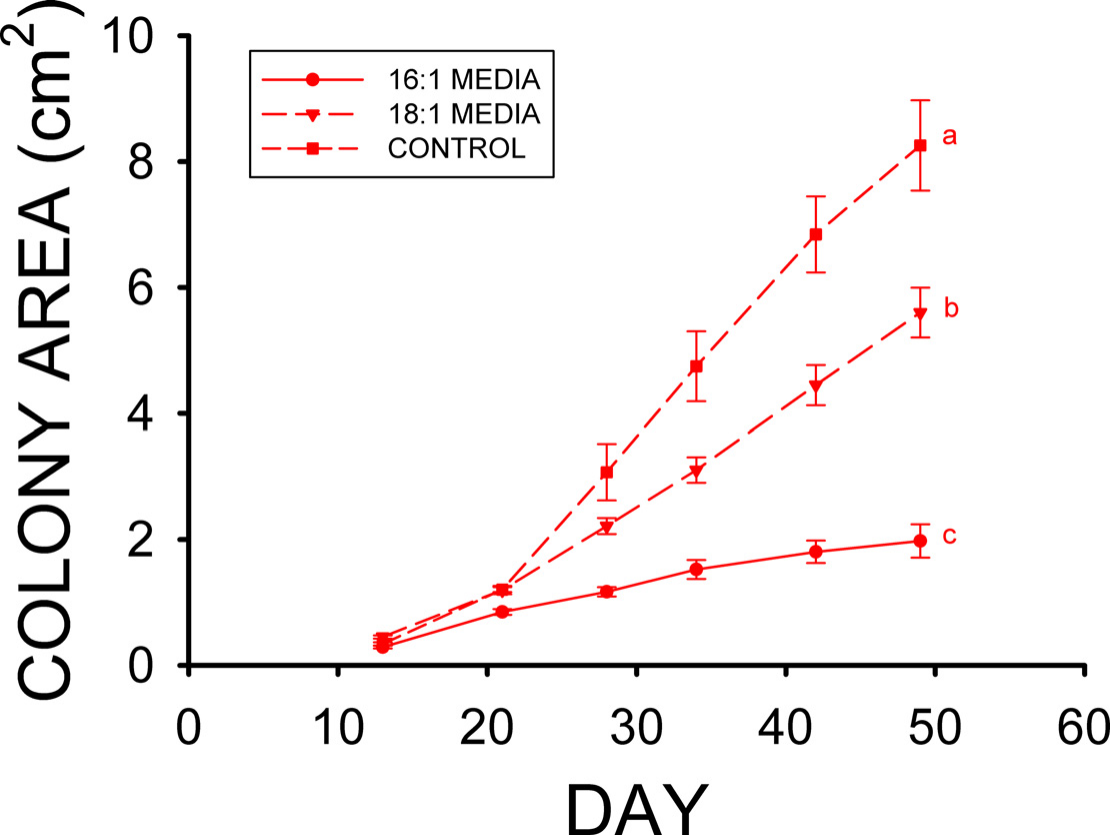
Mean (± SE) surface areas of *Pseudogymnoascus destructans* colonies at various stages of growth on 1% palmitoleic (16:1) acid (circles), 1% oleic (18:1) acid (triangles), and control (squares) SDA media at T_a_ = 10.5°C. Means sharing a common lower-case letter are not significantly different at the p < 0.05 level.

The mean colony area of the 0.5% stearic acid treatment after 55 d at the high T_a_ was greater than the mean colony area of the 0.5% oleic acid treatment (F_2,86_ = 190.695, p < 0.001) in Experiment 2, and the mean colony area of the control treatment was also greater than those of both the 0.5% stearic and 0.5% oleic acid treatments (Fig 2). The mean colony areas of the 0.5% stearic acid and control treatments after 55 d at the low T_a_ were both greater than that of 0.5% oleic acid treatment (F_2,77_ = 33.850, p < 0.001) in Experiment 2 as well, but did not significantly differ from each other (Fig 2). The mean colony area of the control media was about 8 fold greater than that of the 1% palmitoleic (16:1) acid media treatment (Fig 3) at the end of the third experiment (F_2,57_ = 45.688, p < 0.001), and nearly twice the mean colony area observed on the 1% oleic (18:1) acid treatment. The mean colony area for the 1% oleic acid treatment was also significantly greater than that for the 1% palmitoleic acid media (Fig 3) at the of the third experiment as well. This experiment was conducted at the high T_a_ only, however, due to limitations in the amount of palmitoleic acid that could be obtained.

**Fig 4:**
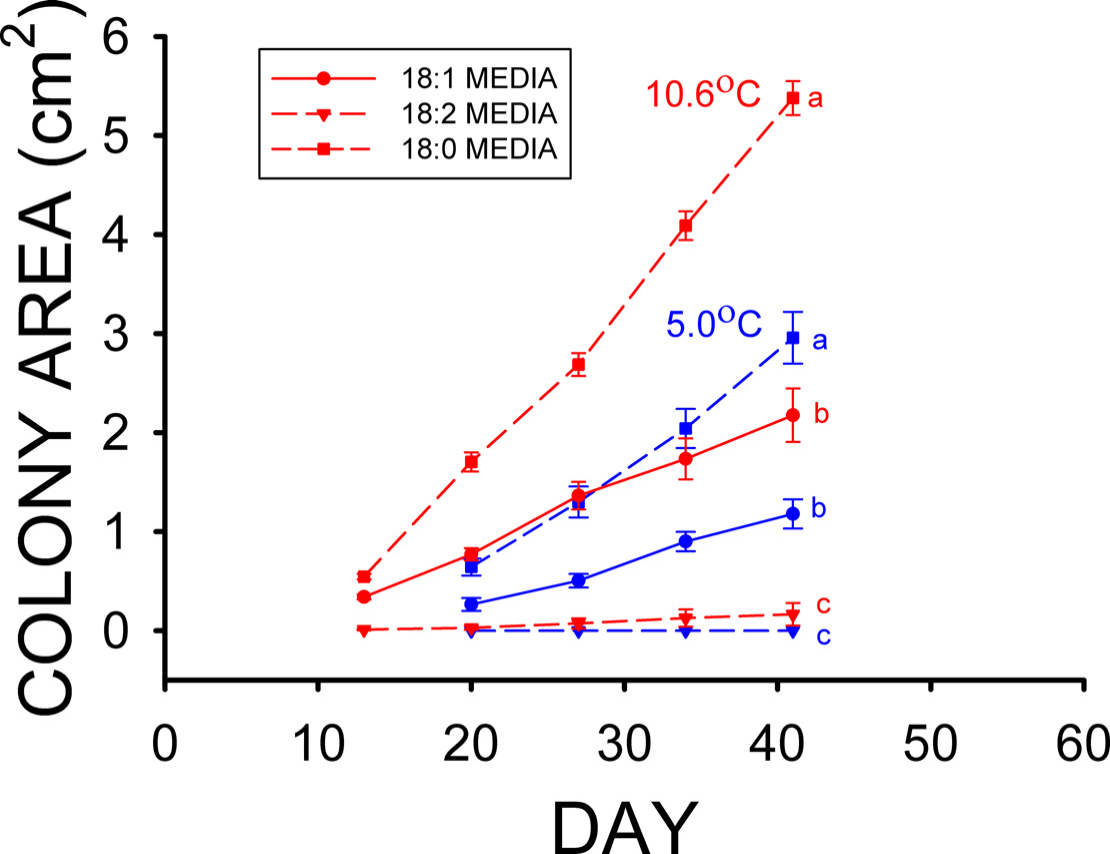
Mean (± SE) surface areas of *Pseudogymnoascus destructans* colonies at various stages of growth on 1% oleic (18:1) acid (circles), 1% linoleic (18:2) acid (triangles), and 1% stearic (18:0) acid (squares) SDA media at T_a_ = 5.0 (blue symbols) and 10.6°C (red symbols). Means within the same T_a_ treatment sharing a common lower-case letter are not significantly different at the p < 0.05 level.

**Fig 5:**
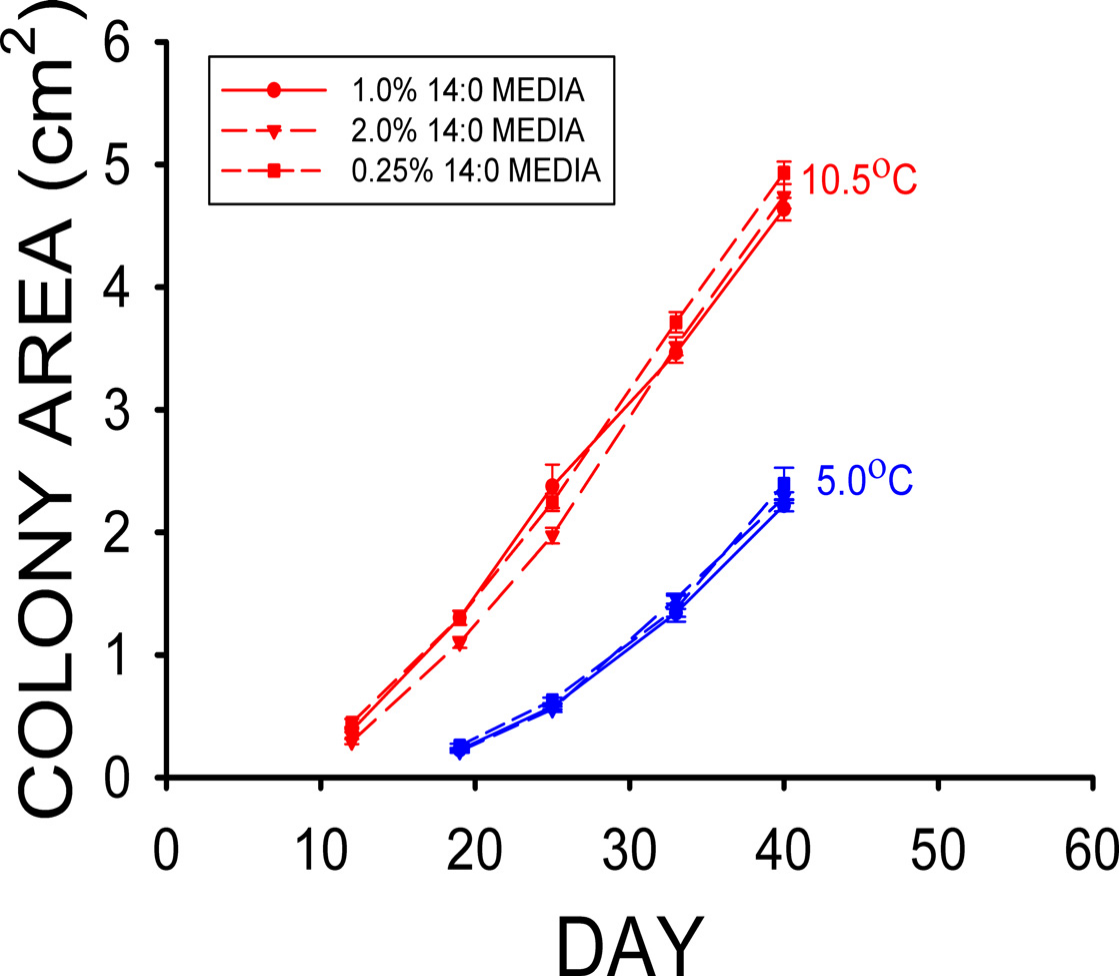
Mean (± SE) surface areas of *Pseudogymnoascus destructans* colonies at various stages of growth on 1% myristic (14:0) acid (circles), 2% myristic acid (triangles), and 0.25% myristic acid (squares) SDA media at T_a_ = 5.0 (blue symbols) and 10.5°C (red symbols). Means within the same T_a_ treatment sharing a common lower-case letter are not significantly different at the p < 0.05 level.

The mean colony area of the 1% stearic acid treatment after 41 d at the high T_a_ was much greater (Fig 4) than those of the 1% oleic and 1% linoleic (18:2) acid treatments (F_2,77_ = 208.64, p < 0.001) in Experiment 4. The mean colony area of the 1% oleic acid treatment was also greater than that of the 1% linoleic acid treatment at this point as well (Fig 4). The mean colony area of the 1% stearic acid treatment after 41 d at the low T_a_ was substantially greater than those for both the 1% oleic and 1% linoleic acid treatments (Fig 4), and the mean colony area of the 1% oleic acid treatment was also significantly greater than that of the 1% linoleic acid treatment (F_2,85_ = 123.58, p < 0.001) of Experiment 4. The mean colony areas of the 0.25, 1.0, and 2.0% myristic acid treatments were not significantly different from each other in both the high T_a_ (F_2,87_ = 2.66, p = 0.08) and low T_a_ (F_2,87_ = 1.18, p = 0.31) groups (Fig 5) at the end of Experiment 5.

## Discussion

The triacylglycerols, diacylglycerols, wax esters, and trace amounts of glycerophospholipids found in the mammalian stratum corneum are all esters containing fatty acids [38], thus epidermal fatty acids exist in both free and ester-bound forms. Our analyses measured the total amount of each fatty acid type found in both the free and ester-bound forms, thus the fatty acid compositions reported in Tables 2 and 3 represent the total amount of each fatty acid present in both forms. The total amount of each fatty acid type present in both the free and ester-bound forms will affect the growth of *Pd* on a substrate because fungi use lipases and phospholipases to invade host tissues. Fungal lipases catalyze the hydrolysis of triacylglycerols, diacylglycerols, and wax esters to monoacylyglycerols, FFAs, and glycerol, thereby releasing FFAs. Likewise, phospholipases hydrolyze 1 or more ester linkages in glycerophospholipids, releasing FFAs as well [39]. A recent study demonstrated that *P*. *destructans* secretes the same lipases and phospholipases as other species of fungi [40], consequently the growth of *Pd* on a substrate causes the conversion of fatty acids bound to triacylglycerols, waxes, and glycerophospholipids to FFAs.

The results of our *Pd* culture experiments support our hypothesis that some of the fatty types found in the wing epidermis of bats inhibit the growth of *Pd*, and they also indicate that the differences in epidermal fatty acid composition observed can have profound influences on *Pd* growth. Experiment 1 clearly demonstrates that myristic acid significantly reduces the growth of *Pd* at T_a_ = 13.4°C (Fig 1), whereas the growth of *Pd* at 10.5°C is also reduced by the addition of stearic acid to the media in Experiment 2 (Fig 2). Neither of these two saturated fatty acids influenced growth of *Pd* at T_a_ = 4.0–5.0°C, however. The results of experiments 3 and 4 demonstrate that the two monounsaturated fatty acids found in the wing epidermis of both bat species, oleic and palmitoleic acids, each greatly reduce the growth of *Pd* (Figs 3 and 4). Oleic acid reduced *Pd* growth at both 5 and 10.5°C (Figs 3 and 4). Linoleic acid was the only polyunsaturated fatty acid found in the wing epidermis of *M*. *lucifugus* and *E*. *fuscus* (Table 2). Experiment 4 demonstrates that linoleic acid almost completely inhibits the growth of *Pd* at both 5.0 and 10.5°C (Fig 4). The results of Experiment 5 reveal that concentrations of 0.25 to 2.0% FFA are equally effective at reducing the growth of *Pd* (Fig 5) at T_a_ = 5–10.5°C. The relative levels of *Pd* growth inhibition by the addition of either myristic, palmitoleic, stearic, oleic, or linoleic acid to the Sabouraud dextrose agar (SDA) media can be determined by comparing the mean *Pd* colony areas obtained by the end of Experiments 1, 2, 3 and 4 to those for the control (SDA only) cultures grown at the same T_a_ in each experiment. The mean colony area for the 0.5% myristic acid treatment was 94% of that for the control media at high T_a_ (Fig 1), by the end of Experiment 1, whereas the mean colony area of the 0.5% stearic acid treatment in the high T_a_ group of Experiment 2 was 89% of that for the control media after 48 d (Fig 2). The mean colony area of the 1% oleic acid treatment in the high T_a_ group of Experiment 3 was 68% of the mean colony for the corresponding control plates after 49 d (Fig 3), whereas the mean colony area of the 1% palmitoleic acid treatment was just 24% of control levels by this point. The mean colony area of the 1% linoleic acid treatment after 41 d of growth at the high T_a_ (Fig 4) was just 1–2% of the control plates for high T_a_ groups of Experiments 1, 2, and 3. The relative ability of these fatty acids to inhibit the growth of *Pd* at T_a_ = 10.5–13.4°C is thus: 14:0 ≈ 18:0 < 18:1 < 16:1 < 18:2. At T_a_ = 4.0–5.0°C, neither myristic nor stearic acids reduce the growth of *Pd*, but the results of Experiments 2 and 4 demonstrate that oleic and linoleic acids both greatly reduce *Pd* growth in this T_a_ range, with the effect of linoleic acid again being much greater than that of oleic acid. Interestingly, the addition of palmitic (16:0) acid to the SDA media actually enhanced to growth of *Pd* (Fig 1) at both T_a_ ranges, relative to the controls.

These observed effects of various FFAs on the growth of *Pd* are largely consistent with the antifungal activities previously observed for these fatty acids against other species of fungi. Myristic, palmitoleic, oleic, and linoleic acids all have been shown to inhibit the growth of numerous fungal species other than *Pd*, whereas palmitic acid does not inhibit the growth of some fungi [41, 42]. The profound reduction of *Pd* growth caused by the addition of linoleic acid to the media is of particular significance because mammals can synthesize either saturated or monounsaturated fatty acids, but they are incapable of producing polyunsaturated fatty acids (PUFAs). Most plant species, in contrast, produce two types of PUFAs: linoleic acid (18 carbons, 2 double bonds) and α-linolenic acid (18 carbons, 3 double bonds). When mammals ingest dietary lipids, however, any PUFAs consumed are incorporated into their membrane and storage lipids [38]. All bats in temperate regions are insectivorous [9–11], thus the linoleic acid found in the skin of both *E*. *fuscus* and *M*. *lucifugus*, is derived from their insect diet. Insect species themselves vary in their ability to synthesize PUFAs; some cannot synthesize either linoleic or α-linolenic acids, whether other species are able to synthesize linoleic acid. The PUFA content of insects therefore varies with both species and their diets [43, 44].

The wing epidermis of *M*. *lucifugus* and *E*. *fuscus* did not significantly differ in linoleic acid content (Table 2) during the first month hibernation. The wing sebum collected from 13 bat species was analyzed for fatty acid content by Pannkuk *et al*. [45], and the sebum of *M*. *lucifugus* was found to contain significantly: a) more stearic acid, and, b) less oleic acid than the wing sebum from *E*. *fuscus*. These findings are consistent with the results of our study, and indicate that the species differences we observed in epidermal stearic and oleic acid levels are due, at least in part, to corresponding differences in sebum composition.

The linoleic acid content of wing epidermis from *M*. *lucifugus* decreased by about half during the course of the hibernation period (Table 3), in contrast, and myristic and stearic acid levels decreased as well. These changes in epidermal fatty acid content may explain why severe cutaneous *Pd* infections are not normally observed in this species until about the middle of the hibernation period [18]. The changes in cutaneous fatty acid content that occur during the course of the hibernation period, and their potential effects on *Pd* growth in the epidermis, both warrant further investigation.

Differences in: a) the ability of some saturated fatty acids to inhibit the growth of *Pd* at low temperatures, b) epidermal palmitoleic acid content, and, c) epidermal oleic acid content, each may contribute to the ability of *E*. *fuscus* to better resist cutaneous *Pd* infections than *M*. *lucifugus*. At temperatures of 10.5–13.4°C, myristic and stearic acids moderately reduce the growth of *Pd* to approximately the same extent, but neither influence the growth of *Pd* at 4.0–5.0°C, as stated previously. The combined (14:0 + 18:0) levels of these 2 saturated fatty acids in the epidermis of both *M*. *lucifugus* and *E*. *fuscus* are similar (Table 2) at the onset of hibernation. It is unlikely that the presence of myristic and stearic acids present in the epidermis of *M*. *lucifugus* confers any resistance to *Pd* infection however, since the T_skin_ of this bat species is normally 5–7°C during natural hibernation [35]. The T_skin_ of hibernating *E*. *fuscus*, in contrast, is usually 12–13°C [19]. Consequently, the myristic and stearic acids in *E*. *fuscus* epidermis can contribute to the ability of this species to resist *Pd* infections. The mean palmitoleic and oleic acid contents of *E*. *fuscus* epidermis were almost twice that of *M*. *lucifugus* at the onset of hibernation (Table 2), and this may also be one of the factors that enable *E*. *fuscus* to better resist cutaneous *Pd* infections since these fatty acid are potent inhibitors of *Pd* growth. Our findings thus support the hypothesis that the wing epidermis of bat species susceptible to cutaneous infection with *Pd* have relatively lower contents of the fatty acids that inhibit the growth of this fungus than that of bat species that are resistant to *Pd*. Further investigation of the role of cutaneous lipids in the susceptibility of bats to cutaneous *Pd* infections will therefore provide novel and important insights into the potential spread of WNS in North America.
